# RETRACTED: Chou et al. Muscle Damage and Performance After Single and Multiple Simulated Matches in University Elite Female Soccer Players. *Int. J. Environ. Res. Public Health* 2021, *18*, 4134

**DOI:** 10.3390/ijerph22121821

**Published:** 2025-12-05

**Authors:** Tai-Ying Chou, Kazunori Nosaka, Trevor C. Chen

**Affiliations:** 1 Department of Athletic Performance, National Taiwan Normal University, Taipei City 11677, Taiwan; tychou1963@gmail.com; 2 Department of Physical Education and Sports Science, National Taiwan Normal University, Taipei City 11677, Taiwan; 3 Centre for Exercise and Sports Science Research, School of Medical and Health Sciences, Edith Cowan University, Joondalup, WA 6027, Australia; k.nosaka@ecu.edu.au

The journal retracts the article titled “Muscle Damage and Performance after Single and Multiple Simulated Matches in University Elite Female Soccer Players” [1], cited above.

Following publication, concerns were brought to the attention of the Editorial Office regarding potential irregularities in the research protocol and informed consent information presented in this publication [1].

Adhering to our standard procedure, an investigation was conducted by the Editorial Office and Editorial Board, with input from the National Taiwan Normal University, that confirmed that this study does not adhere to MDPI’s Research Involving Human Subjects policy (https://www.mdpi.com/ethics#_bookmark9). As a result, the Editorial Board has decided to retract this publication [1], as per MDPI’s retraction policy (https://www.mdpi.com/ethics#_bookmark30).

This retraction was approved by the Editor-in-Chief of the journal *International Journal of Environmental Research and Public Health*.

Tai-Ying Chou did not provide a comment on this decision. Kazunori Nosaka and Trevor C. Chen agreed to this retraction.
